# Supplementary figures and images for: Restriction of S-adenosylmethionine conformational freedom by knotted protein binding sites
Source: PLoS Comput Biol. 2020 May 26;16(5):e1007904. doi: 10.1371/journal.pcbi.1007904 (PMC7319350; doi:10.1371/journal.pcbi.1007904)

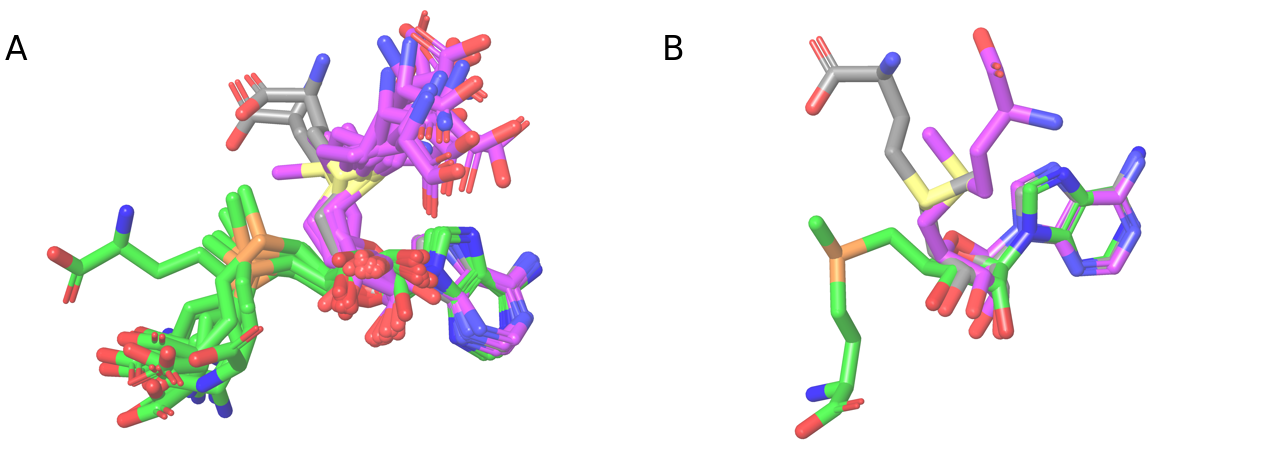

Supplement: S1 Fig — Panel A shows extended conformations from unknotted MTs (green), bent SAMs from knotted MTs (purple), and rare conformations from knotted MTs with extended methionine moiety (grey). Panel B depicts one structure from each of these groups. (TIF) [file pcbi.1007904.s001.tif]

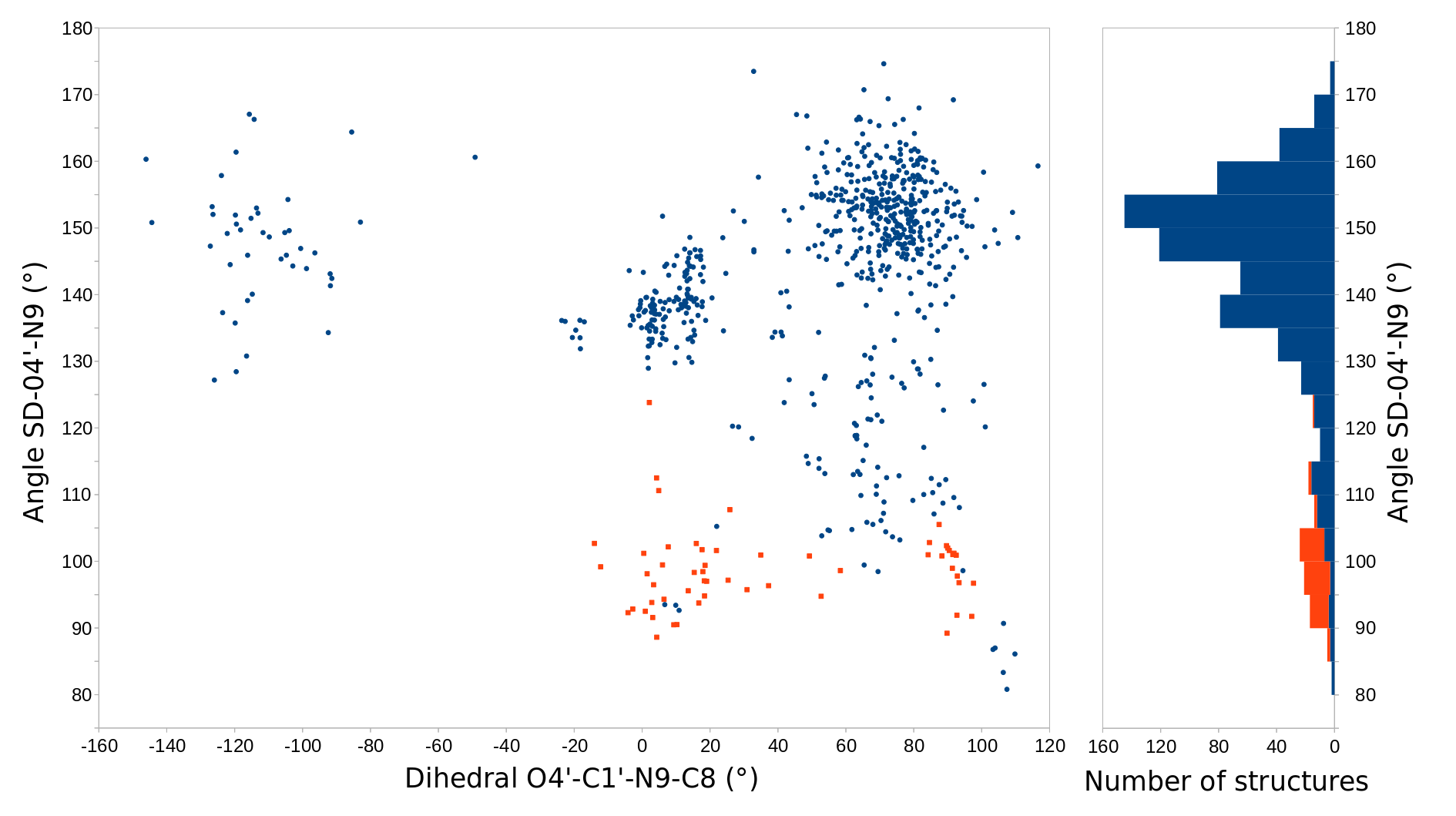

Supplement: S2 Fig — The right panel: distributions of angle SD-O4’-N9. (TIF) [file pcbi.1007904.s002.tif]

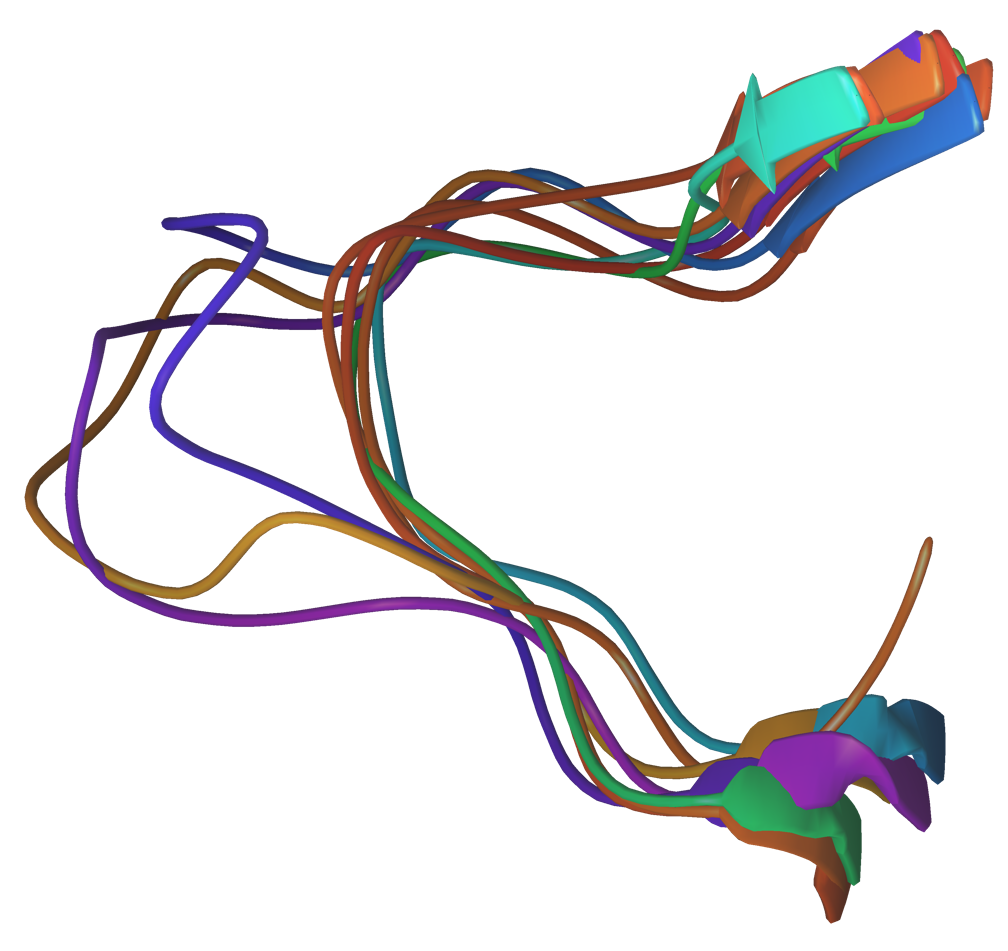

Supplement: S3 Fig — Superimposed on SAM adenine moiety’s heavy atoms. (TIF) [file pcbi.1007904.s003.tif]

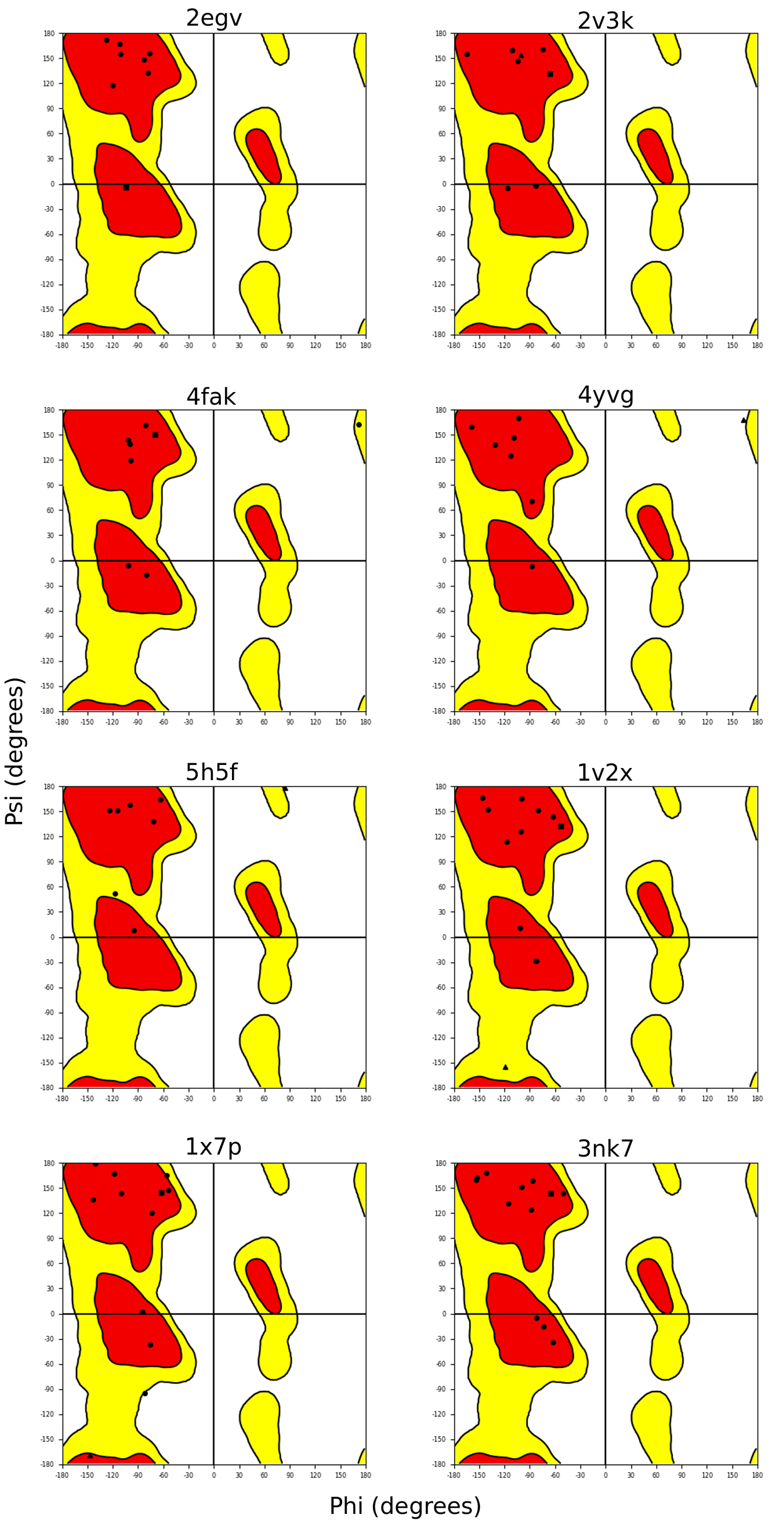

Supplement: S4 Fig — (TIF) [file pcbi.1007904.s004.tif]

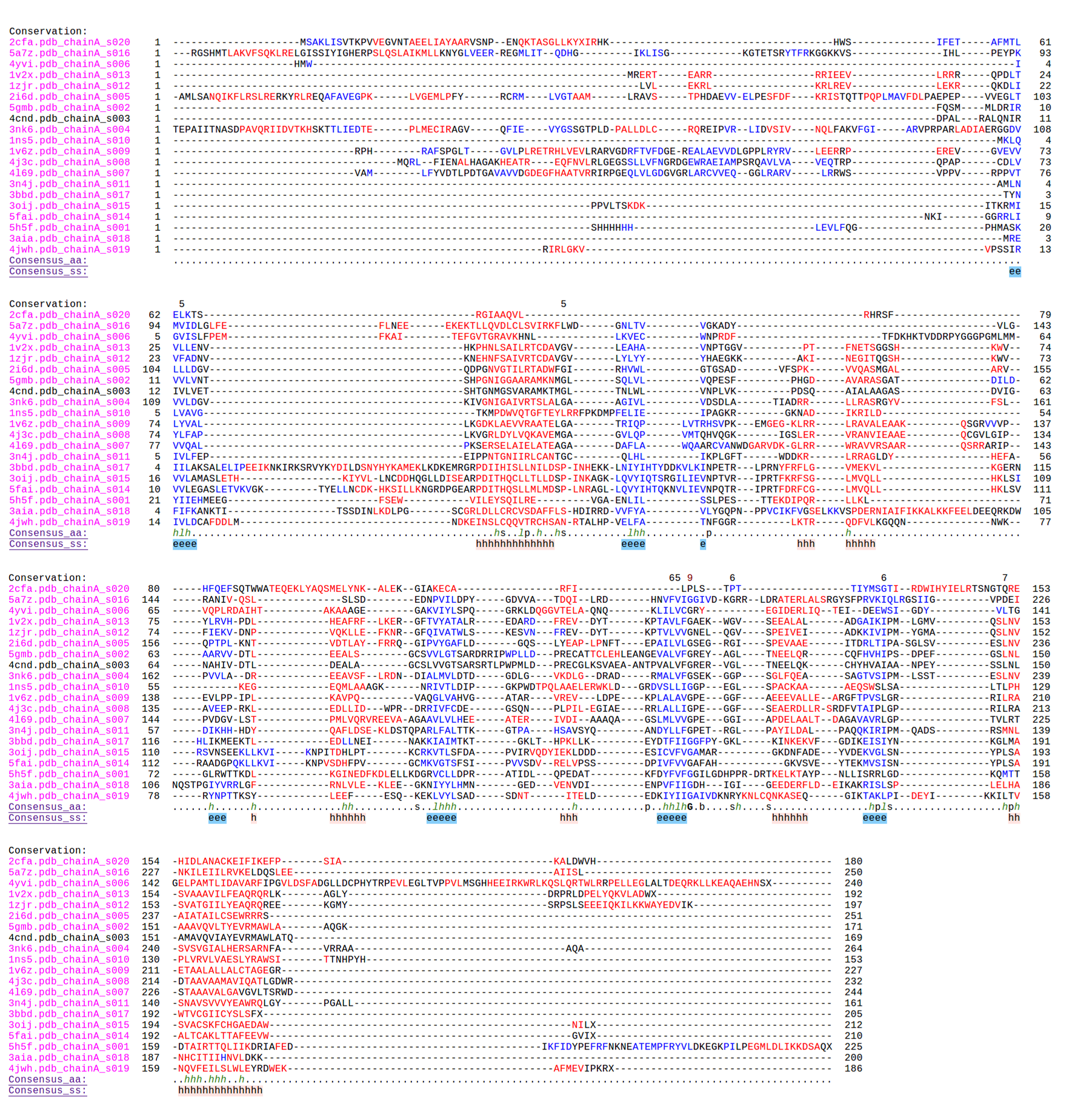

Supplement: S5 Fig — (TIF) [file pcbi.1007904.s005.tif]

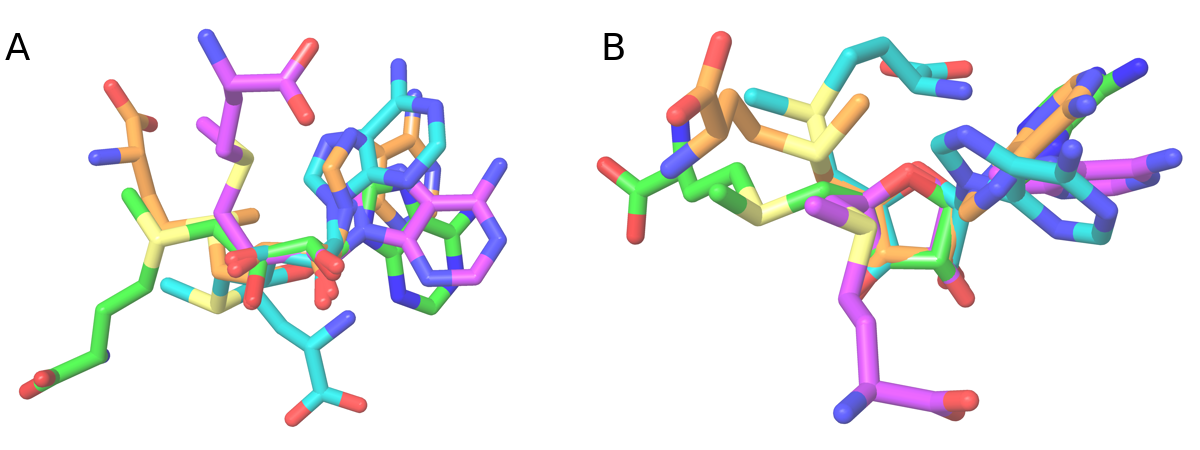

Supplement: S6 Fig — Green: unknotted protein (PDB ID: 4dmg); purple: knotted MT (PDB ID: 4yvg); orange: knotted SAM synthase (PDB ID: 4ndn); teal: unknotted histone MT (PDB ID: 1n6c). A: side view; B: view from the top. (TIF) [file pcbi.1007904.s006.tif]

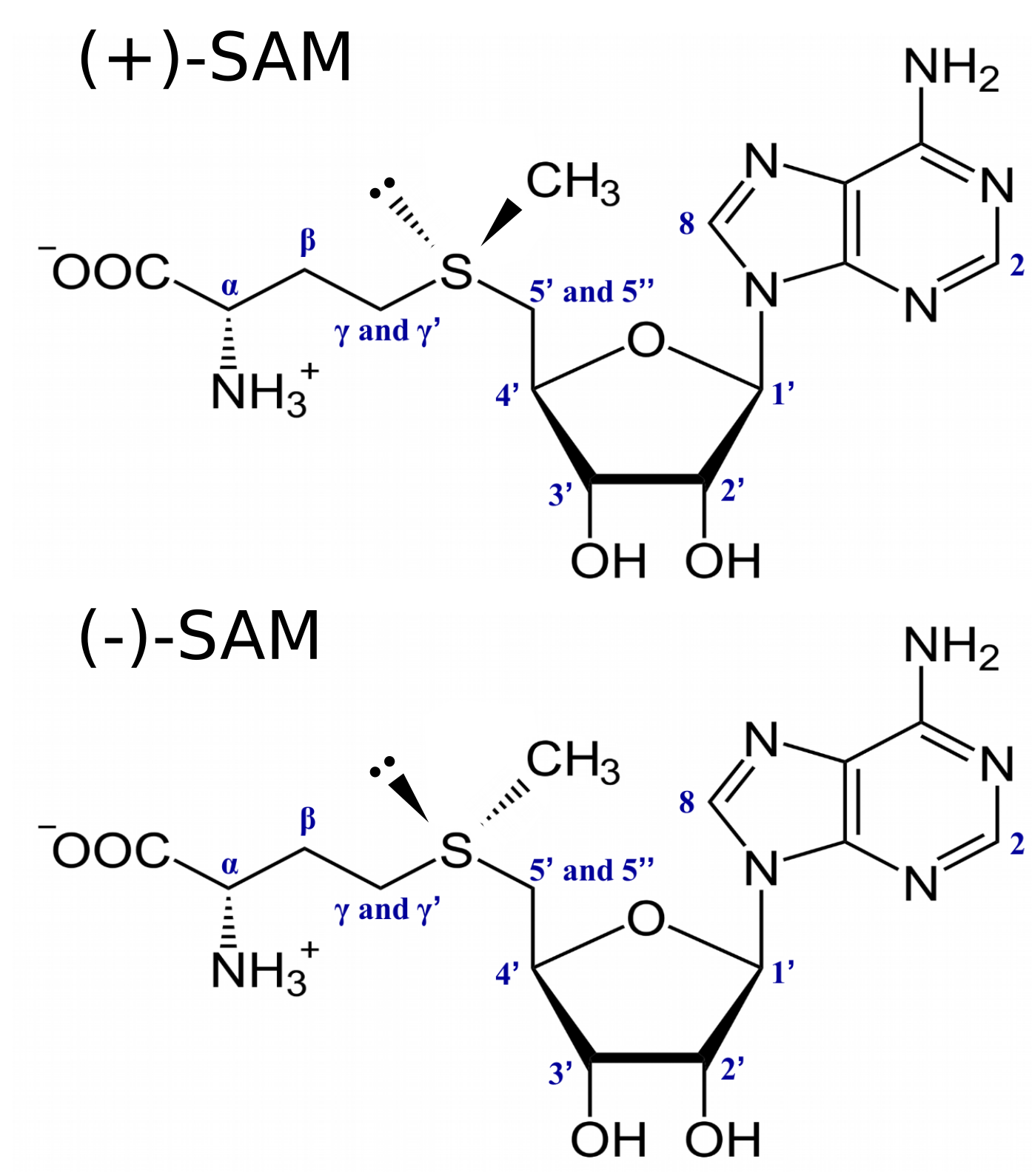

Supplement: S7 Fig — (TIF) [file pcbi.1007904.s007.tif]

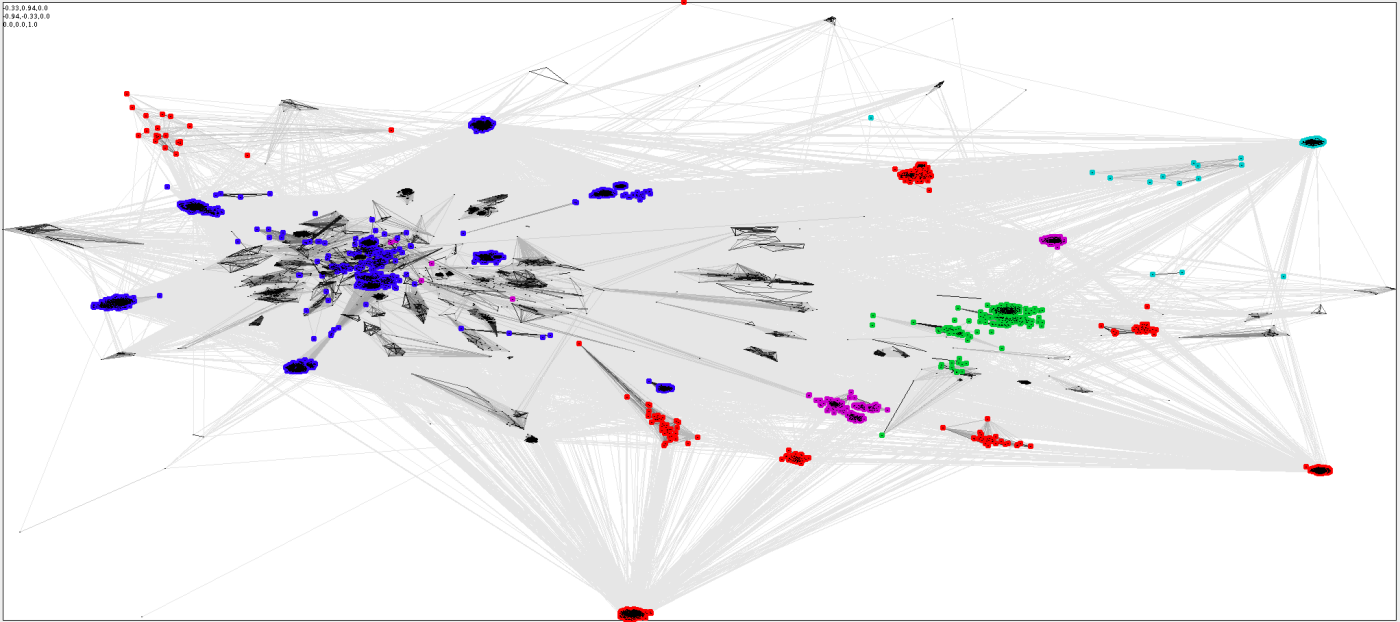

Supplement: S8 Fig — Rossmann Fold (Class I; blue), TIM beta/alpha barrel (Class II; cyan), tetrapyrrole MTs (Class III; purple), SPOUT (Class IV; red), SET domain (Class V; green). Black color refers to unannotated methyltransferases. The proteins with similarity threshold (P) lower than 10−25 are joined by lines, which are darker the greater the similarity. The proteins belonging to each class are separated from other classes and are forming smaller groups, which shows that sequential differences in SAM-dependent MTs are present between as well as within each class. (TIF) [file pcbi.1007904.s008.tif]
