# Supplementary material for: Restriction of S-adenosylmethionine conformational freedom by knotted protein binding sites
Source: PLoS Comput Biol. 2020 May 26;16(5):e1007904. doi: 10.1371/journal.pcbi.1007904 (PMC7319350; doi:10.1371/journal.pcbi.1007904)
Supplement: S1 Table — (PDF) [file pcbi.1007904.s009.pdf]

Gene/protein name	PDB ID	Resolution [Å]	Species	Ligand	RNA type
TrmD	1uak	2.05	Haemophilus influenzae	SAM	tRNA
RsmE	2egv	1.45	Aquifex aeolicus	SAM	rRNA
AviRb	1x7p	2.55	Streptomyces viridochromogenes	SAM	rRNA
Tsr	3gyq	2.45	Streptomyces azureus	SAM	rRNA
Trm56	2yy8	2.48	Pyrococcus horikoshii	SAM	tRNA
TrmY	3ai9	1.55	Methanocaldococcus jannaschii	SAM	rRNA
rlmH/YbeA	4fak	1.7	Staphylococcus aureus	SAM	rRNA
TrmH	1v2x	1.5	Thermus thermophilus	SAM	tRNA
TrmJ	4cng	1.1	Sulfolobus acidocaldarius	SAH	tRNA
TrmL	4jal	2	Escherichia coli	SAH	tRNA
TARBP1/Trm3	2ha8	1.6	Homo sapiens	SAH	tRNA
Nep1	3oin	1.9	Saccharomyces cerevisiae	SAH	rRNA
