# Supplementary material for: Restriction of S-adenosylmethionine conformational freedom by knotted protein binding sites
Source: PLoS Comput Biol. 2020 May 26;16(5):e1007904. doi: 10.1371/journal.pcbi.1007904 (PMC7319350; doi:10.1371/journal.pcbi.1007904)
Supplement: S2 Table — (PDF) [file pcbi.1007904.s010.pdf]

Gene/protein name	PDB ID	Resolution [Å]	Species	Ligand	RNA type
Trm10	4jwf	2.4	Schizosaccharomyces pombe	SAH	tRNA
Trm10a	4fmw	2	Homo sapiens	SAH	tRNA
Sfm1	5h5f	1.7	Saccharomyces cerevisiae	SAM	tRNA
