# Supplementary material for: Restriction of S-adenosylmethionine conformational freedom by knotted protein binding sites
Source: PLoS Comput Biol. 2020 May 26;16(5):e1007904. doi: 10.1371/journal.pcbi.1007904 (PMC7319350; doi:10.1371/journal.pcbi.1007904)
Supplement: S3 Table — (PDF) [file pcbi.1007904.s011.pdf]

Gene/protein name	PDB ID	Resolution[Å]	Quaternary structure	Species	Ligand	RNA type
TrmI	1i9g	1.98	monomer, creates tetramers	Mycobacterium tuberculosis	SAM	tRNA
rlmA	1p91	2.8	dimer	Escherichia coli	SAM	rRNA
rlmE	1eiz	1.7	monomer	Escherichia coli	SAM	rRNA
rlmO	4dmg	1.7	dimer	Thermus thermophilus	SAM	rRNA
RlmCD	5xj2	2.84	monomer	Streptococcus pneumoniae	SAH	rRNA
RlmD (rumA, ygcA)	2bh2	2.15	monomer	Escherichia coli	SAH	rRNA
RsmC	3dmf	1.58	monomer	Thermus thermophilus	SAM	rRNA
taw2	3a25	2.3	monomer	Pyrococcus horikoshii	SAM	tRNA
Trm8	2vdv	2.3	monomer	Saccharomyces cerevisiae	SAM	tRNA
Trm61	5ccb	2	tetramer	Homo sapiens	SAH	tRNA
RsmA (KsgA)	3ftf	2.8	monomer	Aquifex aeolicus	SAH	rRNA
TrmU54	2jjq	1.8	monomer	Pyrococcus abyssi	SAH	tRNA
ermC	1qao	2.7	monomer	Bacillus subtilis	SAM	rRNA
PAPS	1vpt	1.8	dimer	Vaccinia virus	SAM	mRNA
CMTR1	4n48	2.704	monomer	Homo sapiens	SAM	mRNA
RsmG (gidB)	3g89	1.5	monomer	Thermus thermophilus	SAM	rRNA
Trm1	3axt	2.491	dimer	Aquifex aeolicus	SAM	tRNA
Trm14	3tm4	1.95	monomer	Pyrococcus furiosus	SAM	tRNA
RlmJ	4blv	2	monomer	Escherichia coli	SAM	rRNA
PPM2 (TYW4)	2zw9	2.5	monomer	Saccharomyces cerevisiae	SAM	tRNA
