# Supplementary material for: Restriction of S-adenosylmethionine conformational freedom by knotted protein binding sites
Source: PLoS Comput Biol. 2020 May 26;16(5):e1007904. doi: 10.1371/journal.pcbi.1007904 (PMC7319350; doi:10.1371/journal.pcbi.1007904)
Supplement: S4 Table — The calculation is based on the Principal Component Analysis of MD simulations of protein-SAM-tRNA complexes. Entropy of SAM A from TrmD and SAM from Trm5 are statistically different (p-value = 0.0052; Student’s t-test). (PDF) [file pcbi.1007904.s012.pdf]

	TrmD		Trm5
	SAM A	SAM B	SAM
Traj1	30.41	28.45	44.60
Traj2	29.02	55.79	42.62
Traj3	25.66	21.01	29.51
Traj4	31.93	33.61	34.41
Traj5	26.59	31.20	40.65
Traj6	30.12	27.21	35.00
Traj7	28.84	28.26	33.12
Traj8	27.24	29.53	24.84
Traj9	32.76	32.46	24.54
Traj10	-	-	61.84
Average	29.17	31.95	37.11
Standard Error	0.80	3.22	3.50
