# Supplementary material for: Restriction of S-adenosylmethionine conformational freedom by knotted protein binding sites
Source: PLoS Comput Biol. 2020 May 26;16(5):e1007904. doi: 10.1371/journal.pcbi.1007904 (PMC7319350; doi:10.1371/journal.pcbi.1007904)
Supplement: Attachment — Submitted filename: PLoS report.pdf [file pcbi.1007904.s013.pdf]

The general scope of this study is to contribute in addressing the problem whether native knots which are present in about 2% of the proteins in the PDB database have a functional role related to binding. In particular, in this work the authors focus on binding between knotted methyltransferases (MTs) and S-adenosylmethionine (SAM) enzymes. The main reason for this choice is that knotted and unknotted MTs bind their SAM substrate in different manners.

To address this problem, the authors employ a variety or different experimental and theoretical techniques in the attempt of exploring the conformational space of SAMs, while in isolation, and while binding to knotted and unknotted MTs.

After defining suitable structural descriptors (e.g. a collection of specific dihedral angles) they compare fits of experimental NMR data, PDB structures and relatively long MD simulations for characterizing the differences for SAM conformations in isolation and in complex with knotted and knotted MT.

Their first finding is that, not surprisingly, unbound SAMs have larger conformational freedom than in complex.
A slightly less expected finding is the evidence of a significant difference in the SAM conformation in complex with a knotted and unknotted MT proteins. Based on the results of mutagenic experiments the authors suggest this conformation may be biologically active.

This study is conducted in a rather methodologically sound and systematic way. Overall, the results are discussed and reported in a clear and exhaustive manner.

However I think the authors should elaborate further on their finding about structural differences of SAM binding to knotted and unknotted MT proteins. In particular, the paper would be much improved if they quantitively addressed the question whether the structural changes in the binding motif to knotted and unknotted are significantly larger than those observed comparing to SAM binding to all different unknotted MTs. In other words, I would like the authors to provide analyses to exclude that the structural differences in the binding motifs that they have found is not trivially a consequence of the differences in the Mts primary structures.

Overall, I find it very difficult to draw any biological conclusions about the functional role of protein knots from the data reported in this work. It is of course possible (and perhaps useful) to speculate on the functional role of the specific conformational changes they induce. But I think that, overall, this work does not bring any new and conclusive solid evidence in support of functional role of knotting.

On the other hand,, this paper does provide a detailed characterization of these specific structural differences induced in knotted and unknotted proteins. This information may in principle be useful when attempting to design more specific anti-bodies.
In addition, this work might motivate further studies aiming to find out if some of the structural motifs associated to binding to knotted sites found here are conserved throughout epigenetically and structurally diverse families of knotted proteins.

Based on these considerations, I think this work provides sufficient new results to

deserve publication on PLoS Comp. Biology.
