# Supplementary material for: Restriction of S-adenosylmethionine conformational freedom by knotted protein binding sites
Source: PLoS Comput Biol. 2020 May 26;16(5):e1007904. doi: 10.1371/journal.pcbi.1007904 (PMC7319350; doi:10.1371/journal.pcbi.1007904)
Supplement: Attachment — Submitted filename: letter_8.pdf [file pcbi.1007904.s014.pdf]

Dear editors,
Thank you for the effort in editing our manuscript and we thank both reviewers for their constructive comments about our work. We apologize for the late reply, but we wanted to thoroughly respond to comments and add additional data. Below we state point-by-point response to all remarks stated (editor's and referees' comments are in blue).

Reviewer #1:
This is an interesting paper that examines the binding of the common cellular cofactor S-adensoylmethionine (SAM) to two different classes of methyltransferase (MTase), one class containing a trefoil knot topology, the other being unknotted. In addition to comparing both SAM binding modes to the conformation of free in aqueous solution determined using NMR and MD techniques. The aim of the study is to a) identify if there are significant differences in the ligand-binding pockets and b) to understand more about the role of the knot in this class of knotted protein. The analysis of structures from the PDB is rigorous, clearly described and substantial differences in the SAM binding mode detected. The research increases our understanding of the different way SAM can be bound by knotted and unknotted MTases and, in particular, may be very important for the rational design of selective inhibitors.

Thank for the appreciation of our work, we are glad to read this.

However, there are a number of ways in which the paper does not provide an adequate view of protein binding sites in general, and it does not cite some of the key papers in the field that highlight what is known about the folding of knotted proteins and that, to some degree, question the basic premise regarding the conservation of knotting in proteins. The authors need to address to the following points:
Introduction p. 1. The term "unorthodox" is used to describe the binding mode of SAM in knotted MTases. However, no analysis of the other SAM-binding proteins is given only. It may well be that the bent configuration adopted by knotted MTases is also adopted by many other SAM binding proteins.

Thank you for pointing this. The term unorthodox may be ambiguous. Conformations of SAM in knotted MTs are indeed unique. However, their specificity lies not only in being bent. Although rare, there are also bent SAM conformations in unknotted proteins and in knotted non-MT proteins, as we described in our paper. SAM conformations in knotted MTs are unorthodox, because of the combination of being bent and having specific range of the glycosidic dihedral angle at the same time.

p2 and throughout the manuscript. The trefoil knot in knotted MTases is referred to as tight throughout the manuscript. This is an incorrect use of this term. Many groups have shown using AFM and optical tweezers that a tightened trefoil knot comprises between 12-13 amino acid residues. The trefoil know in native MTases spans 45 amino acid residues. The authors are really referring to the length of the stretch of peptide that contains the knot. The wording needs to be changed throughout the manuscript as it is currently misleading.

Thank you; indeed there is a big difference between the size of the knot when stretched and in the native state. In the paper we refer to the native state of a not stretched knot. Since this was not clear to the reviewer we modified this sentence. We also added some examples of proteins with a large/broad knot, which composed almost all structure of the protein and point out readers to KnotProt where the histogram of a knot core in the non-redundant set of proteins is shown.

P2. "The ligand-binding cavity is embedded inside this knot core". This might be misleading as it suggests that only the knot core region participates in SAM binding. This is not correct. There are also important contacts outside of the knot core region and these need to me mentioned.

We agree with the Reviewer that this statement might be misleading. The knot is only a part of the SAM binding site, although in this case it is one of the most important parts. We implemented necessary changes in the manuscript.

P2 and 3. Paragraph starting "Moreover,…..". There are a number of key points to make about this paragraph. First of all, there are a large number of examples of where differently built proteins perform the exact same function - it is called convergent evolution. At the very least, an additional sentence is needed to alert the reader to the fact that this is not the only example that Nature has come up with. Second, with reference to the sentence that knotted proteins are more difficult to fold than there unknotted counterparts. This statement is incorrect in the cellular context in which these proteins actually fold. It has been clearly shown for some time that the folding of this class of knotted MTase is fast in the presence of molecular chaperones. I am surprised that this paper which came out in 2012 has not been cited. It should also be made clear that many unknotted proteins fold very slowly and that this is not a property of knotted proteins alone.

According to the first remark: we agree with that comment. We changed the beginning of the paragraph to more explicit, emphasizing that this is an example. Also, we added additional points to show the reader that knotted proteins may be a special example of convergent evolution, as they seem to be less likely "picked" by the evolution.

According to the second remark. Here we refer to experimental data for knotted and unknotted protein (King NP, Jacobitz AW, Sawaya MR, Goldschmidt L, Yeates TO. Structure and 543 folding of a designed knotted protein. Proceedings of the National Academy of 544 Sciences. 2010;107(48):20732-20737.). We agree with the reviewer that the presence of molecular chaperons significantly speed up folding of YbiK and YbeA, both proteins are members of Spout superfamily (Mallam AL, and Jackson SE, Knot formation in newly translated proteins is spontaneous and accelerated by chaperonins. Nat Chem Biol. 2011 Dec 18;8(2):147-53.). We are happy to cite this paper (Mallam AL, and Jackson SE, Nat Chem Biol. 2011), however according to our knowledge there is no data about direct comparison of knotted and unknotted member of Spout in the

experimental approach. There is a suggestion from numerical simulation that Yibk fold significantly faster than YibK without knot (Sulkowska JI, Sulkowski P, Onuchic J. Dodging the crisis of folding proteins with knots. PNAS. 2009;106(9):3119-3124.).
We agree with the fact, that many unknotted proteins fold very slowly and also there are many proteins, which fold much faster and in general such proteins can be compared. Moreover, the general folding of knotted proteins is still not fully understood. However, this topic is out of the scope of this paper. Therefore, herein we focus just on the members of Spout superfamily and we will point out readers to current reviews about folding of knotted proteins.
We modified this paragraph and added additional references.

P6/7 Some more detail on how active and inactive conformations of SAM binding should be given.

The data regarding biological activity of SAM conformations is limited - there are only two different structures of the whole protein complexes (including the substrate) of knotted MTs. However, we elaborated further the aspects of ligand's bent and extended methionine moiety conformations and their active/inactive status based on the current knowledge.

P^. Reference to "tight" cavities and conformational freedom. Conformational freedom is referred to frequently but the analysis presented in the paper does not provide any information on conformational dynamics. The fact that the cluster of SAM conformations in the knotted MTAses is lower than in the unknotted MTase does not imply anything about the dynamics. Additional analysis/experiments are required in order to be able to make any statement. For example, B-factors from the crystal structures could be used as an indirect measure of dynamics, MD simulations could be performed, NMR experiments directly measuring dynamics could be used. The fact that there is a larger distribution of conformations of SAM bound to unknotted Mtases may simply be a consequence of this family being represented by a larger number of structures in the PDB or the sequence similarity of this family of unknotted MTases being lower.

We are sincerely grateful for this reviewer's insight! Following the suggestions we conducted extensive explicit solvent MD simulations of whole complexes for two analogous MTs - TrmD (knotted) and Trm5 (unknotted), which included SAM and the substrate (tRNA). Based on these data we calculated descriptors, including angle distributions, which allowed us to observe greater conformational freedom of SAM in the Trm5 binding site, especially in terms of the glycosidic dihedral angle. Also, we compared the dynamics of the ligands in their respective binding sites based on calculated RMSF (Figure 9) and on the configurational entropy (Table S4). Overall, the new MD simulations confirm more quantitatively our previous implications based on the PDB data.

Additionally, during implementing data gathered from new MD simulations, we found an inaccuracy in the division of proteins to knotted and unknotted. Very limited portion of knotted proteins was incorrectly classified as unknotted. Thus, we remade the

distributions of both angles for the SAM conformations from PDB. As only single proteins were inaccurately classified, the new results do not differ much from the previous ones, and the conclusions remain the same. We added these results to the paper.

To visualize the importance of angle distributions for distinguishing knotted and unknotted proteins, we added a scatter plot (Figure S2) of plain and dihedral angle of SAM conformations from PDB in the Supplementary Materials, clearly showing that angle SD-O4'-N9 is a good descriptor of the protein's entanglement.

P9. Last paragraph the term well-preserved should be replaced with highly conserved.

We agree with the Reviewer that the term "well-preserved" is unsuitable, and we replaced it according to the suggestion.

P13. The authors may want to be aware that there are other examples for unknotted proteins where circular permutants affect activity/function.

Thank you for pointing this out, we found additional data (Pandey, N., Kuypers, B. E., Nassif, B., Thomas, E. E., Alnahhas, R. N., Segatori, L., & Silberg, J. J. (2016). Tolerance of a knotted near-Infrared fluorescent protein to random circular permutation. Biochemistry, 55(27), 3763-3773.), however, if there are more such examples, we will greatly appreciate more detailed suggestion.

Reviewer #2:
The general scope of this study is to contribute in addressing the problem whether native knots which are present in about 2% of the proteins in the PDB database have a functional role related to binding. In particular, in this work the authors focus on binding between knotted methyltransferases (MTs) and S-adenosylmethionine (SAM) enzymes. The main reason for this choice is that knotted and unknotted MTs bind their SAM substrate in different manners.

To address this problem, the authors employ a variety or different experimental and theoretical techniques in the attempt of exploring the conformational space of SAMs, while in isolation, and while binding to knotted and unknotted MTs.

After defining suitable structural descriptors (e.g. a collection of specific dihedral angles) they compare fits of experimental NMR data, PDB structures and relatively long MD simulations for characterizing the differences for SAM conformations in isolation and in complex with knotted and knotted MT.

Their first finding is that, not surprisingly, unbound SAMs have larger conformational freedom than in complex.
A slightly less expected finding is the evidence of a significant difference in the SAM conformation in complex with a knotted and unknotted MT proteins. Based on the results of mutagenic experiments the authors suggest this conformation may be biologically

active.

This study is conducted in a rather methodologically sound and systematic way. Overall, the results are discussed and reported in a clear and exhaustive manner.

However I think the authors should elaborate further on their finding about structural differences of SAM binding to knotted and unknotted MT proteins. In particular, the paper would be much improved if they quantitively addressed the question whether the structural changes in the binding motif to knotted and unknotted are significantly larger than those observed comparing to SAM binding to all different unknotted MTs. In other words, I would like the authors to provide analyses to exclude that the structural differences in the binding motifs that they have found is not trivially a consequence of the differences in the Mts primary structures.

Due to the fact, that SAM-dependent methyltransferases are a vast group of distinctly built proteins, that vary sequentially even within one class, it is hard to quantitively assess the differences between them. For example, both the knotted and the Rossmann Fold MTs have highly structurally conserved core that creates the majority of the ligand binding site. In both cases it is invariant in only few positions. Moreover, it was shown that there is no sequence similarity between these proteins (Kozbial, P. Z., & Mushegian, A. R. (2005). Natural history of S-adenosylmethionine-binding proteins. BMC structural biology, 5(1), 19.). Nevertheless, we performed a clustering of the sequences of all available SAM-dependent methyltransferases (almost 11 000 proteins) to show that they divide in several sequentially distinct groups, even within single class (Figure S8). Therefore, the differences in the binding motif of SAM are a result of both the sequential and the structural differences between the proteins. However, in addition to our current results we decided to compare the glycine motif common for the methyltransferases' binding sites and show that the way it is resolved depends on the protein fold (Figure 11).

Overall, I find it very difficult to draw any biological conclusions about the functional role of protein knots from the data reported in this work. It is of course possible (and perhaps useful) to speculate on the functional role of the specific conformational changes they induce. But I think that, overall, this work does not bring any new and conclusive solid evidence in support of functional role of knotting.

We agree with the Reviewer that drawing biological conclusions about the role of protein knots might be exaggerated. We made necessary changes in the manuscript and toned down these statements.

On the other hand,, this paper does provide a detailed characterization of these specific structural differences induced in knotted and unknotted proteins. This information may in principle be useful when attempting to design more specific anti-bodies.
In addition, this work might motivate further studies aiming to find out if some of the structural motifs associated to binding to knotted sites found here are conserved throughout epigenetically and structurally diverse families of knotted proteins.

Based on these considerations, I think this work provides sufficient new results to deserve publication on PLoS Comp. Biology.

We thank the reviewer for positive comments on our work. We've corrected all of the suggested fragments, which improved overall the manuscript.

Additionally, as suggested by the editor, we included the Ramachandran plots of the adenine-binding loops of the representative knotted MTs (with previous reference "data not shown") in the Supplementary Materials (Figure S4).

Once again, we thank both referees and the editor for the effort made in judging our manuscript and for all the comments. We hope, that after the corrections we applied, our manuscript is suitable for high standards of PloS Comp. Biology.

Sincerely,
Joanna Sulkowska
