# Supplementary material for: Restriction of S-adenosylmethionine conformational freedom by knotted protein binding sites
Source: PLoS Comput Biol. 2020 May 26;16(5):e1007904. doi: 10.1371/journal.pcbi.1007904 (PMC7319350; doi:10.1371/journal.pcbi.1007904)
Supplement: Attachment — Submitted filename: review_response_27_03_2020(1).docx [file pcbi.1007904.s015.docx]

Reviewer #1: I have read through the authors response to the reviewers
comments in detail and can see that they have acknowledged the issues
that the two reviewers had with the manuscript, which is excellent.
HOWEVER, when I go to read the revised manuscript some of the changes
that it was claimed were undertaken have clearly not been. I don't know
why this is. For example, just on page 2 alone, line 15, the knot is
still referred to incorrectly as tight. Line 19, it is still said that
the ligand binding site is embedded within the knot core making no
references to contacts outside of this region. Line 29, again the knot
is referred to as tight. I have not read the whole manuscript because I
do not feel it is appropriate for a reviewer to have to carefully go
through a revised manuscript that the authors stat has been revised
according to the reviewers comments when it has not been. I am happy to
read through an additional revised manuscript where the revisions have
been made.

We apologize for the misunderstanding. We felt that the changes we made were sufficient. Regarding the term “tight”, we changed it to “compact” to avoid confusion. We also added the information to emphasize that not only knot is involved in ligand binding.

We have carefully read through the manuscript to check for other inconsistencies. We hope that you will find our changes satisfactory.

Reviewer #2: The authors have answered all comments in a satisfactory
way and improved their manuscript accordingly. I recommend publication
as is.

Thank you. We greatly appreciated all the Reviewer’s comments and questions. They considerably contributed to the improvement of our manuscript.
